# Hand and forearm immersion in hot water at half-time enhances subsequent leg muscle strength

**DOI:** 10.1055/a-2605-0149

**Published:** 2025-10-10

**Authors:** Yuto Yamashita, Yoshihisa Umemura

**Affiliations:** 1 Graduate School of Health and Sport Sciences, Chukyo University, 101 Tokodachi, Kaizu-cho, Toyota, Aichi, Japan

**Keywords:** core temperature, muscle strength, half-time, recorvery, passive heat Maintenance

## Abstract

We aimed to investigate the effects of hot-water immersion of the hand and forearm during half-time (HT) on the physiological responses, leg muscle strength, and cycling sprint performance in the cold. Ten recreationally active men performed the experimental trials that consisted of 40 min intermittent cycling, followed by a 15-min HT, and then an intermittent cycling sprint test in a cold (5°C and 50% relative humidity). During HT, the participants underwent two different interventions: seated rest (CON) or hand and forearm heating (HEAT). The intermittent cycling sprint test comprised 10 sets of 5 s of maximal pedaling and 25 s of recovery. In addition, the participants performed maximal voluntary contraction (MVC) in knee extension before and after HT. Although the peak power output in the intermittent cycling sprint test did not significantly differ between trials (p>0.05), the rectal temperature (T _re_ ) following HT in HEAT was significantly higher than in CON (p=0.026). In addition, the MVC force after HT was significantly higher in HEAT than in CON (p<0.001). This suggests hot-water immersion of hand and forearm during HT improves knee extensor muscle strength and attenuates the T _re_ decrease in a cold environment.

## Introduction

Team sports such as rugby and soccer are played throughout the year, and therefore it is not uncommon for matches to be played in a cold environment. It is important for the players of such sports to be able to perform high-intensity exercise in the form of running and sprinting 1 . However, several studies have shown that high-intensity exercise performance declines early in the second half of matches 2 3 . Therefore, sports scientists are interested in strategies that could be implemented at half-time (HT) to prevent this decline in high-intensity exercise performance.

One of the reasons why high-intensity exercise performance declines early in the second half of matches is a lack of physical preparation, owing to the rest taken during HT 4 . This resting is associated with physiological changes, such as decreases in core and muscle temperature, metabolic rate, and muscle activation 5 6 . In soccer matches, it has been shown that players’ core and muscle temperatures decrease by 1.1°C and 2.0°C, respectively, during HT, and that the decrease in muscle temperature during HT correlates with the subsequent decrease in sprint performance (r=0.60) 4 . Moreover, the reduction in core temperature measured during a simulated 15-min HT was found to correlate with the decline in peak power output during a subsequent countermovement jump (CMJ) (r=0.63) in professional rugby union players 7 . Therefore, the decrease in body temperature during HT may underpin the decline in exercise performance after HT.

In team sports, re-warm-up (RW) and passive heat maintenance are often implemented as an HT strategy 8 . However, RW can only be performed for a short period of time, usually<3 min, owing to the time constraints of HT 9 . It has been reported that a high-intensity, short-duration RW is not sufficient to prevent the decrease in core temperature that occurs during HT, although it does improve subsequent high-intensity exercise performance in a thermoneutral environment 5 . Thus, short-duration RW may not effectively counteract the reduction in body temperature that occurs at HT in a cold environment, when core and muscle temperature may substantially decrease during resting, unlike in a thermoneutral environment.

Several studies have shown that passive heat maintenance, achieved by wearing survival or heating garments during HT and following the warm-up period, reduces the size of the decrease in core temperature and improves jump and sprint performance vs. normal training attire in a thermoneutral environment 7 10 . In addition, passive heat maintenance can be applied for a longer period of time than RW because it can be performed while the player is sitting 8 . In a cool environment (8°C, 50% relative humidity (RH)), the core temperature during 25 min of passive rest remains significantly higher and the subsequent time taken to complete a 2,000-m rowing course is superior when an external heating garment is worn during 25 min of passive rest following a warm-up than if a standard tracksuit top is worn during this period 11 . Thus, passive heat maintenance during HT may reduce the decrease in body temperature and exercise performance and may therefore be a useful HT strategy for players in a cold environment.

Immersion of the whole body and lower limbs in hot water is another passive heating method that is used to increase body temperature and improve physiology 12 13 . It might be that heat is transferred more rapidly between the environment and the body using this method, because the thermal conductivity of water is much higher than that of air (0.6 vs. 0.025 W/mK). However, players may not be able to immerse their whole body and lower limbs in hot water because of the time required to take off and put on shoes and socks. However, the hands have a high surface area-to-mass ratio and contain arteriovenous anastomoses (AVA), which together with the superficial veins between the wrist and the elbow, constitute a specialized heat exchange organ 14 that facilitates substantial heat transfer. Immersion of the hand in hot (or cold) water has been described an efficient rewarming (or cooling) method 15 . A recent study showed cold water immersion of the hand and forearm was effective in decreasing core temperature and improving exercise performance in a hot environment 16 17 . However, no study has evaluated the effect of hand and forearm immersion in hot water during HT on subsequent physiological changes and exercise performance in a cold environment. Hot-water immersion of the hand and forearm results in warmed blood returning from the periphery to the core and may therefore reduce the decrease in core temperature during HT in a manner with similar to theories of cold-water immersion. Therefore, in the present study, we aimed to investigate the effect of hot-water immersion of the hand and forearm during HT on subsequent physiological response, leg muscle strength, and cycling sprint performance of volunteers in a cold environment. We hypothesized that hot-water immersion of the hand and forearm during HT would reduce the subsequent decline in core body temperature and improve leg muscle strength and cycling sprint performance.

## Material and Methods

### Participants

Participants were 10 recreationally active men (age [mean±standard deviation (SD)] 22±2 years, height 170.6±2.1 cm, body mass 63.1±4.5 kg, V̇O _2max_ 44.5±4.1 mL·kg ^−1^ ·min ^−1^ ) who habitually played team sports (soccer, lacrosse, or ultimate frisbee) intermittently trained (≥1 h/session) on>2 days per week participated in the study. None of the participants were smokers and none had a history of cardiovascular disease. The study was approved by the University Ethics Committee on Research with Human Subjects (approval number: 2023-020). Before they participated in the study, all participants provided written informed consent.

### Overview of the trial

The participants visited the laboratory four times. During the first visit, they performed a graded exercise test using a cycle ergometer to determine their V̇O _2max_ , then the second visit was a familiarization session. During the third and fourth visits, they participated in two experimental sessions in randomized order that were separated by at least 72 h. Each experimental session consisted of 40 min of intermittent cycling exercise, a 15-min HT, and an intermittent cycling sprint test. During the 15-min HT, the participants either rested in a seated position (CON) or immersed their hand and forearm in hot water (43°C) up to the elbow (HEAT). The experimental sessions were conducted in a climate chamber (TBR12A4PX; ESPEC, Osaka, Japan) set at 5°C and 50% RH and were at the same time of day to minimize the confounding effect of circadian variations in body temperature. During the experimental period, the participants were instructed to maintain their regular lifestyle and level of physical activity and to finish eating at least 3 h before each experimental session. In addition, they were asked to avoid strenuous activity and the intake of caffeine or alcohol for the 24 h preceding each session.

### V̇O _2max_ measurement

The participants performed a maximal graded exercise test on a bicycle ergometer with electromagnetic brakes (Fujin-Raijin; O.C. Labo, Tokyo, Japan) to determine their V̇O _2max_ . After a 3-min warm-up at 100 W, the maximal graded test started at 100 W, and this was increased by 20 W every 2 min until volitional exhaustion 18 . The participants were asked to maintain a cadence of 80 rpm. Their oxygen uptake ( V̇O _2_ ) was analyzed breath-by-breath using an automatic gas analyzer (AE-310s; Minato Medical Science, Tokyo, Japan) and averaged over 30-s periods. V̇O _2max_ was determined when two of the following three criteria were met: 1) there was a plateau in V̇O _2_ , 2) the heart rate of the participant was within 10% of the predicted maximum (220 minus their age in years), and 3) the respiratory exchange ratio was>1.05. The heart rates of the participants were continuously recorded during the test using a heart rate (HR) monitor (Polar A-300; Polar, Kempele, Finland) 19 20

### Experimental protocol

**Fig. 1:**
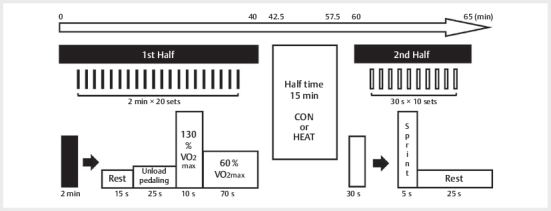
Experimental protocol. CON: control trials, HEAT: hot-water immersion trials.

Upon arrival at the laboratory, the participants’ health and fatigue were assessed verbally and their body mass were measured. After the placement of skin and rectal thermistors, an HR sensor, and a surface electrode, the participants entered the climate chamber, which was set at 5°C and 50% RH. They then rested on a chair for 5 min, after which they performed a warm-up consisting of 1.2 kp with a cadence of 80 rpm for 5 min and 5 s of maximal pedaling against a resistance of 7.5% of body mass 18 . After the warm-up, the participants performed the first half of their exercise, which was composed of 20 2-min exercise periods. The protocol for each experimental session is depicted in Fig. 1 . Each period was composed of 15 s of rest, 25 s of unloading cycling, 10 s of high-intensity cycling (130% of V̇O _2max_ ), and 70 s of moderate-intensity cycling (60% of V̇O _2max_ ). This protocol was designed to reflect activity levels during a soccer match in a previous study. The participants were instructed to maintain a cadence of 80 rpm during the first half of the exercise, except during the 15-s rest period.

Subsequently, during the 15-min HT, the participants either rested (CON) or underwent the heat intervention (HEAT) in a climate chamber. After HT, the participants performed an intermittent cycling sprint test, which was composed of 10 sets of 5 s of maximal pedaling against a resistance of body weight×0.075 kp and 25 s of active recovery, to evaluate their cycling sprint performance. This intermittent cycling sprint protocol was modified from a previous study that investigated intermittent cycling sprint performance 21 22 .The participants were asked to stop pedaling for 5 s before pedaling at maximum speed from a stationary start. The maximal voluntary isometric contraction (MVC) force during the extension of the right knee was measured before HT and after HT. Before the first half of the exercise protocol, participants performed MVC as a familiarization. In addition, the neuromuscular activity of the left vastus lateralis was measured during MVC. We measured the MVC force and neuromuscular activity within 2.5 min of the start and end of HT.

### Heating intervention

During the HEAT session, each participants immersed their hand and forearm into a constant-temperature water tank at 43°C (T-3MD; Thomas Kagaku, Tokyo, Japan) while sitting on a chair for 15 min. During the hot-water immersion, the participants wore a sleeveless T-shirt (made of 100% polyester), standard tracksuit bottoms (made of 100% polyester), and a long down coat (made of 100% polyester). The participants in the CON group wore a long-sleeved T-shirt and a standard tracksuit top (made of 100% polyester) in addition to the clothes worn during the HEAT session and rested on the chair for 15 min.

### Measurements

#### Performance index

The peak power outputs of the participants during each 5-s period of maximal pedaling during the intermittent cycling sprint test were measured using a cycle ergometer (Fujin-Raijin; O.C. Labo) with a sampling frequency of 10 Hz.

The MVC force associated with right knee extension was measured before the first half of the exercise protocol, before HT, and after HT. The measurement was conducted twice and we recorded the higher value of the two obtained as the MVC force. The participants sat in a chair with their hips and knees at angles of 90° and MVC was measured during knee extension over 5 s using a tension meter attachment (T.K.K.1269 f; Takei Scientific Instruments Co. Ltd., Niigata City, Japan) and an analog-to-digital converter (Power Lab; AD Instruments, Bella Vista, New South Wales, Australia) at a sampling frequency of 1,000 Hz. We recorded the MVC forces using LabChart Reader v8.1.5 software (ADInstruments, Sydney, Australia). Only for the measurements made before the first half of the exercise, the participants were required to warm up by exercising at 30%, 50%, and 90% of their MVC. The MVC force before HT was regarded as the baseline value (100%), and the MVC force after HT is expressed as the change in MVC (∆MVC%). Each of the MVC measurements was completed within 2.5 min.

#### Physiological indices

Rectal temperature (T _re_ ) was measured using a rectal thermistor probe (LT-ST08-11; Gram Corporation, Saitama, Japan) that was inserted approximately 15 cm beyond the anal sphincter. Skin temperature was measured using a skin thermistor (LT-ST08-12; Gram Corporation) that was attached to the skin surfaces of the chest (T _chest_ ), arm (T _arm_ ), and thigh (T _thigh_ ). The rectal and skin temperature data were collected using an LT-8 device (Gram Corporation) at 30-s intervals. The mean change in rectal temperature during HT was calculated as ∆T _re_ , and the mean skin temperature ( _m_ T _sk_ ) was calculated using the following formula 23 : 

_m_ T _sk_ =0.43T _chest_ +0.25T _arm_ +0.32T _thigh_

The surface electromyography (EMG) signal from the right vastus lateralis during MVC was recorded using a surface electrode (FA-DL-141; 4Assist, Tokyo, Japan), with an inter-electrode distance of 1.2 cm. The surface electrode was placed over the middle of the muscle belly 24 , and the skin surface was abraded and cleaned to reduce the impedance associated with the skin–electrode interface. The position of the surface electrode was marked with a surgical marker and the electrode was accurately replaced between the two experimental sessions. The EMG signal was recorded using a sampling frequency of 1,000 Hz and an analog-to-digital converter (PowerLab; AD Instruments) and was then band-pass filtered between 20 and 500 Hz using LabChart Reader v 8.1.5 software. The integrated EMG (iEMG) was calculated during the 1-s period that included the maximal MVC force. The iEMG before HT was used as the baseline value (100%), and the iEMG after HT is expressed as the change in iEMG (∆iEMG%). Because of a technical problem, the EMG signal was captured only for nine of the participants.

The HR of each participant was recorded every 1 s using a heart rate monitor (Polar A-300; Polar Electro Oy, Kempele, Finland) and then averaged over 30-s periods.

The blood lactate concentration of each participant was measured in a finger-prick sample collected 1, 3, and 5 min after the intermittent cycling sprint test using a lactate analyzer (Lactate Pro 2 LT-1730; ARKRAY, Kyoto, Japan). The peak lactate concentration (La _peak_ ) was defined as the highest value recorded after the intermittent cycling sprint test.

### Perceptual index

A rating of perceived exertion (RPE; 15-point scale) was provided by each participant using the Borg scale 25 , thermal sensation (TS) was rated using a nine-point scale (1: very cold, 2: cold, 3: cool, 4; slightly cool, 5: neutral, 6; slightly warm, 7: warm, 8: hot, 9: very hot) 26 , and thermal comfort (TC) was rated using a seven-point scale (1: very uncomfortable, 2: uncomfortable, 3: slightly uncomfortable, 4: neutral, 5: slightly comfortable, 6: comfortable, 7: very comfortable). These ratings were provided before the first half, at the end of the first half, at HT, and before and after the second half of the exercise protocol.

### Statistical analysis

All data are presented as mean±SD. Data normality was tested using the Shapiro–Wilk test, and where there was a significant violation of sphericity, F values were adjusted using Greenhouse–Geisser. Peak power output, the rectal and skin temperatures, ∆MVC force, ∆iEMG, HR, RPE, and TS were analyzed using two-way repeated-measures analyses, with session and time as the variables. Post-hoc analyses were performed using the Bonferroni test when a significant interaction was identified. ∆T _re_ and La _peak_ were analyzed using paired *t* -tests. According to Cohen’s d, the effect size (ES) was calculated and assessed as small (0.2–0.5), moderate (0.5–0.8), or large (>0.8). Data were regarded as statistically significant when *p* <0.05.

## Results

### Exercise performance

**Fig. 2:**
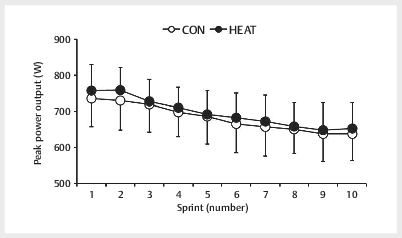
Peak power output during 5 s of maximal pedaling within an intermittent cycling sprint test. Data are mean±SD (n=10). CON: control trials, HEAT: hot-water immersion trials, HT: half-time.

**Fig. 3:**
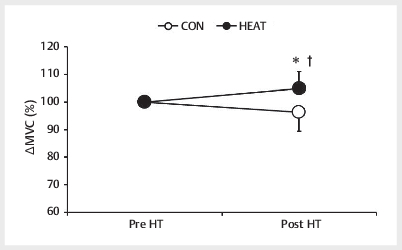
Maximal voluntary isometric contraction (MVC) forces during the extension of the right knee pre- and post-HT. Data are mean±SD (n=10). CON: control trials, HEAT: hot-water immersion trials, HT: half-time. *p<0.05 vs. CON. † p<0.05 vs. pre-HT.

There was a no significant interaction between trial and time with respect to peak power output during the 5 s of maximal pedaling within the intermittent cycling sprint test (F [9,81]=0.788, *p* >0.05), and no significant main effect of the trial (F [1,9]=2.753, CON: 682±72 W, HEAT: 696±64 W, *p* =0.13) ( Fig. 2 ).There was a significant interaction between trial and time with respect to MVC force (F [1,8]=28.789, *p* <=0.001). Furthermore, the MVC force after HT during the HEAT trial was significantly larger than before HT and significantly larger than that during the CON trial (CON: 96.2%±6.9%, HEAT: 105.0%±6.1%, *p* <0.001, ES=1.35) ( Fig. 3 ).

### Rectal and skin temperatures

**Fig. 4:**
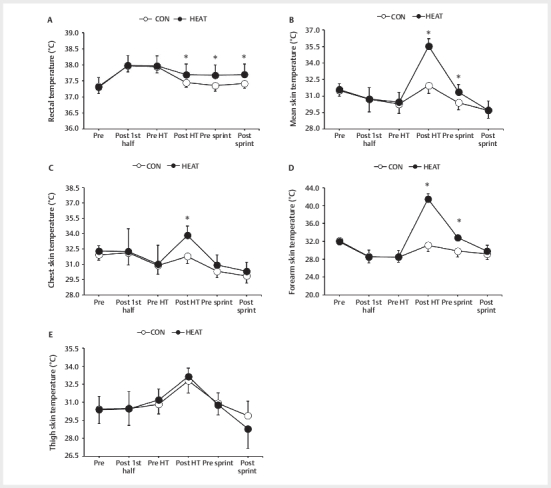
Changes in rectal temperature ( **A** ), mean skin temperature ( **B** ), chest skin temperature ( **C** ), forearm skin temperature ( **D** ), and thigh skin temperature ( **E** ) during the trials. Data are mean±SD (n=10). CON: control trials, HEAT: hot-water immersion trials, HT: half-time. *p<0.05 vs. CON.

There were significant interactions between trial and time with respect to T _re_ , _m_ T _sk_ , T _chest_ , and T _arm_ . (T _re_ : F [5,45]=22.955, *p* <0.001, _m_ T _sk_ : F [5,45]=51.726, *p* <0.001, T _chest_ : F [5,45]=6.018, *p* =0.013, T _arm_ : F [5,45]=114.603, *p* <0.001). There was no interaction between trial and time with respect to T _thigh_ (F [4,45]=2.818, *p* =0.08, Fig. 4E ). The T _re_ ( Fig. 4A ) during the HEAT trial between the post-HT (ES=0.98) and post-sprint (ES=1.09) time points was significantly higher than that during the CON trial ( *p* <0.05). The ∆T _re_ was significantly smaller during the HEAT trials than during the CON trial (CON:−0.50±0.08°C, HEAT:−0.27±0.14°C, *p* <0.001, ES=2.02). The _m_ T _sk_ ( Fig. 4B ) and T _arm_ ( Fig. 4D ) during the HEAT trial at the post-HT ( _m_ T _sk_ : ES=5.18, T _arm_ : ES=8.08) and pre-sprint ( _m_ T _sk_ : ES=1.48, T _arm_ : ES=2.99) time points were significantly higher than during the CON trial ( *p* <0.05). The T _chest_ during HEAT trial at the post-HT time point was significantly higher than during CON trial ( *p* <0.001, ES=1.78, Fig. 4C ).

### Neuromuscular activity

**Fig. 5:**
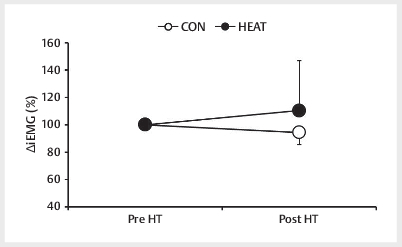
Integrated surface electromyography (iEMG) of the vastus lateralis during measurement of the maximal voluntary isometric contraction (MVC) pre- and post-HT. Data are mean±SD (n=9). CON: control trials, HEAT: hot-water immersion trials, HT: half-time.

There was no significant interaction between trial and time with respect to the change in iEMG (∆iEMG) during MVC (F [1,8]=1.932, *p* >0.05). Therefore, there was no significant difference after HT between the CON and HEAT trials with respect to ∆iEMG (CON: 94.3%±8.9%, HEAT: 110.5%±36.4%, *p* >0.05, ES=0.61) ( Fig. 5 ).

### Heart rate and blood lactate concentration

There was a significant interaction between trial and time with respect to the HR (F [5,45]=11.773, *p* =0.001). The HR at the post-HT in the HEAT trial was significantly higher than that during the CON trial (CON: 84.0±12.1 bpm, HEAT: 101.4±16.9 bpm, *p* =0.001, ES=1.18, Table 1 ). There were no significant differences between the CON trial and the HEAT trial with respect to La _peak_ after the intermittent cycling sprint test (CON: 11.9±2.2 mmol/L, HEAT: 12.4±1.8 mmol/L, *p* =0.205).

**Table:** **Table 1** Heart rate during the trials.

		Pre-1st half	The end of 1st half	Pre-HT	Post-HT	Pre-sprint	Post-sprint
Heart rate (bpm)	CON	86.8±11.0	140.5±7.8	108.3±13.7	84.0±12.1	95.6±11.4	158.0±9.1
	HEAT	83.6±13.9	136.3±10.9	104.2±13.7	101.4±16.9*	102.1±14.6	156.1±9.5

Data are mean±SD (n=10). CON: control trials, HEAT: hot-water immersion trials, HT: half-time. * *p* <0.05 vs. CON.

### Perceptual index

There were significant interactions between trial and time with respect to TS and TC (TS: F [5,45]=8.364, *p* <0.001, TC: F [5,45]=6.319, *p* <0.001) but not with respect to RPE (F [5,45]=0.859, *p* >0.05). The TS during the HEAT trial post-HT ( *p* <0.003, ES=1.19) and pre-sprint ( *p* <0.001, ES=2.07) were significantly higher than those during the CON trial ( Table 2 ). The TC during the HEAT trial pre-sprint was significantly higher than that during the CON trial ( *p* <0.026, ES=1.36) ( Table 2 ). However, there was no significant difference between the CON and HEAT trials with respect to RPE at any time point ( *p* >0.05) ( Table 2 ).

**Table:** **Table 2** Rating of perceived exertion (RPE) and the thermal sensation (TS) and thermal comfort (TC) ratings during the trials.

		Pre-1st half	The end of 1st half	Pre-HT	Post-HT	Pre-sprint	Post-sprint
RPE (6 to 20)	CON	7.3±1.6	13.6±2.2	11.6±3.2	8.5±1.9	8.7±2.2	17.1±1.9
	HEAT	7.6±2.1	13.4±2.2	10.8±2.1	8.3±1.8	8.4±1.7	17.4±1.5
TS (1 to 9)	CON	3.4±0.7	6.7±1.5	5.6±1.7	4.8±1.7	2.9±1.4	5.4±1.1
	HEAT	3.0±1.1	7.0±0.9	6.6±1.3	6.6±1.3*	5.5±1.1*	5.9±1.1
TC (1 to 7)	CON	3.8±1.0	3.6±0.8	4.0±0.9	4.1±1.2	3.4±1.2	3.8±0.6
	HEAT	3.4±0.7	3.5±1.3	4.4±1.2	3.6±1.0	4.9±1.0*	3.5±1.0

Data are mean±SD (n=10). CON: control trials, HEAT: hot-water immersion trials, HT: half-time. * *p* <0.05 vs. CON.

## Discussion

The present study investigated the effect of hot-water immersion of the hand and forearm during HT on subsequent physiological responses, subsequent leg muscle strength, and cycling sprint performance in a cold environment. The principal findings are as follows: hot-water immersion of the hand and forearm during HT attenuated the decrease in T _re_ and _m_ T _sk_ during HT and increased the MVC force post-HT. Moreover, the TS and TC of the participants improved after HT and before a sprint during the HEAT trial. However, although the decrease in T _re_ and _m_ T _sk_ during HT was attenuated by this passive heating, one of the hypotheses could not be proven because the cycling sprint performance of the participants during the HEAT trial was not superior to that during the CON.

The T _re_ post-HT, pre- and post-sprint, and the MVC force post-HT were significantly higher during the HEAT trial than during the CON trial, although the cycling sprint performance of the participants did not significantly differ between the trials. Moreover, during the HEAT trial, the decrease in T _re_ during HT was significantly smaller than that during the CON trial. In the CON trial, the decrease of Tre during HT was –0.50°C±0.08°C. This value was consistent with the decline in T _re_ observed in a previous study 18 during 15-min HT in a cold environment (5°C) following a similar 40-min exercise protocol similar to that in the present study. In the HEAT trial, hot-water immersion of the hand and forearm mitigated the decrease in Tre during HT (∆–0.27°C±0.14°C), whereas a previous study showed high-intensity short-duration RW during HT in a cold environment (5°C) did not mitigate the Tre decrease during HT (∆–0.4°C) 18 . Therefore, passive heat maintenance may be an effective method for mitigating the decrease in core temperature during HT in a cold environment. In addition, wearing a survival jacket during HT in a thermoneutral environment was reported to attenuate the reduction in core body temperature (−0.23°C±0.09°C) 27 , which was a similar change to that observed in the present study. To the best of our knowledge, this is the first study to demonstrate that the decline in core body temperature during HT can be attenuated by hot-water immersion of the hand and forearm, which provides passive heat maintenance in a cold environment. It has been reported that wearing a survival jacket as a passive means of heat maintenance during a simulated HT period by professional rugby union players reduces their decreases in core body temperature (−0.74%±0.08% vs.−1.54%±0.06%), improves their peak power output during CMJ, and improves their repeated sprint ability in a thermoneutral environment 7 . In addition, in a cool environment (8°C, 50%), wearing a passive heating jacket during the transition phase between warming up and the start of competition was shown to increase the core and mean skin temperature and improve 2,000-m rowing performance 11 . Thus, wearing a survival or heating jacket during HT and the transition phase had been previously shown to be effective, but the effects of hot-water immersion had not been assessed. Hot-water immersion has often been used in the past to increase core and muscle temperature 13 28 , and hot-water immersion of the hand and forearm has been suggested to be an effective method of rewarming 15 . The mechanism whereby hot-water immersion attenuates the decline in T _re_ during HT likely involves the AVAs in the arm. Hot-water immersion of the hand and forearm warms the blood in the venous plexus, and this warmed blood returns to the core via superficial veins, which might attenuate the decline in T _re_ during HT.

The peak power output of the present participants during the 5 s of maximal pedaling as part of the intermittent cycling sprint test did not significantly differ between the trials. However, MVC force post-HT was significantly higher than pre-HT in the HEAT trial and post-HT in the CON trial. Although hot-water immersion of the hand and forearm during HT prevented the decline of rectal temperature (∆–0.27°C) in our study, it may not have been sufficient to improve cycling sprint performance in a cold environment. Gavin et al. (2021) reported that wearing an external heating garment during rest periods increased core body temperature (∆0.54°C) and improved subsequent 2000-m rowing performance 11 . Similarly, in a cool or cold environment, external heating methods may be needed to maintain or elevate body temperature and improve high-intensity exercise performance. Moreover, a possible explanation for this may be that MVC measurements were performed before and after HT during both trials. The participants were required to perform the maximal effort for MVC measurements before and after HT, which may have induced fatigue and influenced the peak power output in the subsequent intermittent cycling sprint test. Additionally, cycling sprint performance is influenced not only by body temperature and leg muscle strength but also by energy metabolism and oxygen availability 5 29 . Although gas analysis and muscle oxygenation could not be measured in the present study, hot-water immersion of the hand and forearm may not have improved energy metabolism and oxygen availability, which may explain why improved leg muscle strength did not lead to improved cycling sprint performance. During the HEAT trial, the MVC force post-HT was significantly higher than those pre-HT and at the same time point during the CON trial. This may be important for preventing muscle injury during the start of the second half of a match. Muscle injury risk has been reported to be increased in the start of the second half 30 because of muscle deficiency 30 31 and the decrease in muscle temperature 32 . Therefore, although it cannot be categorically stated based on our results, the improved MVC force we observed after hot-water immersion of the hand and forearm may also contribute to reduced injury risk. An increase in body temperature may improve exercise performance by increasing ATP turnover, the myosin cross-bridge cycling rate, and muscle fiber conduction velocity 33 . Specifically, for every 1°C increase in muscle temperature, exercise performance has been shown to increase by 2%–5% 34 35 , and the decline in core temperature during a simulated HT has been shown to correlate with the decline in peak power output during a subsequent CMJ (r=0.63) 7 . In the present study, although the muscle temperature of the participants were not measured, the T _re_ post-HT during the HEAT trial was significantly higher than that during the CON trial, indicating that the decline in T _re_ was attenuated during the HEAT trial, which may be one of the reasons why the MVC force was higher. Moreover, although the MVC force post-HT was significantly higher during the HEAT trial than during the CON trial, there was no significant difference in the iEMG during MVC between the trials. This may suggest that muscle contractility was improved by the intervention, rather than neuromuscular activity. Increasing muscle temperature by passive heating has previously been shown to improve muscle contractility, which would improve voluntary and involuntary muscle force output 12 13 . Additionally, the performance of both type I and type II muscle fibers is affected by elevations in muscle temperature, although type II muscle fibers are more likely to benefit 33 . Therefore, hot-water immersion of the hand and forearm may have improved type II muscle fiber performance during MVC measurements. Such an improvement in muscle contractility may also explain the improvement in MVC force post-HT. However, a more detailed mechanism for the improvement in MVC force should be established through future studies because muscle temperature was not measured during the present study.

The HR of the participants post-HT was significantly higher during the HEAT trial than during the CON trial. For every 1°C increase in body temperature, HR was shown to increase by approximately 18 bpm because the increase in the excitability of the sinus node 36 . Therefore, the higher HR post-HT during the HEAT trial may be explained by the smaller decline in T _re_ during HT within the HEAT trial than during the CON trial. No difference in the La _peak_ was detected between the trials. The blood lactate concentration is a balance between lactate production and removal. However, aspects of muscle metabolism, such as a muscle oxygenation, were not assessed during the present study, and therefore we cannot discuss lactate production and removal by the participants’ muscles during the intermittent cycling sprint test.

The RPE of the participants did not significantly differ between the trials at any time point, although the T _re_ post-HT and the mean HR during HT were significantly higher during the HEAT trial than during the CON trial. This may indicate that the psychological effects of hot-water immersion during HT are not substantial. In the field, because the implementation of a new HT strategy could interfere with a player’s psychological preparation for the second half of their match 9 , we need to devise a strategy that takes this into consideration. The TS and TC ratings after hot-water immersion of the hand and forearm were superior during the HEAT trial. It has also previously been reported that wearing an external heating garment following a warm-up when in a cool environment (8°C, 50% RH) improves the TS and TC 11 . Thus, although the methods of passive heating in the two studies differed, both improved psychological indices, such as TS and TC. In the present study, the improvements in TS and TC may have been the result of higher mean skin temperatures after the hot-water immersion because ratings of thermal sensation are closely associated with the skin temperature 37 . In addition, it has been suggested that improving TC before exercise may improve subsequent performance 11 . Although intermittent cycling sprint performance was not improved by hot-water immersion in the present study, the psychological responses after HT were improved, and these effects should be further explored in future studies.

### Limitations

The present study had some limitations. First, we simulated the exercise patterns of team sport matches using cycling exercise, whereas such matches typically involve running-based exercise, including jogging, high-intensity running, and jumping, and there are differing metabolic and locomotory responses in people performing cycling- and running-based exercise. Thus, the findings in the present study may be difficult to apply directly to team sports in a field setting. Although the exercise pattern in this study was not similar to actual team sports matches, the exercise duration and the degree of increase in body temperature would be close to the actual match. Therefore, future studies should investigate the effect of hot-water immersion of the hand and forearm in participants performing a running-based exercise protocol. Second, in the present study, the hot-water immersion lasted for 15 min during HT. However, during the HT period in team sports, players typically return to their changing room, undergo injury treatment, participate in tactical debriefing, and change their clothing. Therefore, it has been suggested that HT performance-improving strategies could not last for>9 min 9 . Moreover, although we used water at 43°C, the use of water at a different temperature may yield different effects. Third, post-HT and pre-sprint _m_ T _sk_ were significantly higher in the HEAT trial than in the CON trial. However, _m_ T _sk_ was strongly influenced by forearm skin temperature changes due to hand and forearm immersion in hot water (43°C), which is a limitation of the methodology used. Finally, the peak power output during the 5 s of maximal pedaling during the intermittent cycling sprint test did not significantly differ between the trials. As mentioned above, this may be because MVC was assessed just before the intermittent cycling sprint test, and this may have obscured the effect of hot-water immersion. Our results could also have been influenced by the small sample size. Further studies should use larger sample sizes and modify the experimental protocol to avoid this potential confounder to further investigate the effect of hot-water immersion on intermittent cycling sprint performance.

## Conclusions

The findings of the present study indicate that hot-water immersion of the hand and forearm during a 15-min break in exercise in a cold environment attenuates the decline in core body temperature that occurs during this period and improves the subsequent MVC force, TS, and TC. Thus, hot-water immersion of the hand and forearm during HT may represent a new strategy to improve muscle strength for use in team sports played in a cold environment.
